# Diabetes Mellitus Alters the Immuno-Expression of Neuronal Nitric Oxide Synthase in the Rat Pancreas

**DOI:** 10.3390/ijms23094974

**Published:** 2022-04-29

**Authors:** Bright Starling Emerald, Sahar Mohsin, Crystal D’Souza, Annie John, Hussain El-Hasasna, Shreesh Ojha, Haider Raza, Basel al-Ramadi, Ernest Adeghate

**Affiliations:** 1 Departments of Anatomy, College of Medicine & Health Sciences, United Arab Emirates University, Al Ain P.O. Box 17666, United Arab Emirates; bsemerald@uaeu.ac.ae (B.S.E.); smohsin@uaeu.ac.ae (S.M.); crystal.dz@uaeu.ac.ae (C.D.); 2 Departments of Biochemistry, College of Medicine & Health Sciences, United Arab Emirates University, Al Ain P.O. Box 17666, United Arab Emirates; anniej@uaeu.ac.ae (A.J.); h.raza@uaeu.ac.ae (H.R.); 3 Departments of Medical Microbiology and Immunology, College of Medicine & Health Sciences, United Arab Emirates University, Al Ain P.O. Box 17666, United Arab Emirates; hussain.hasasna@usask.ca (H.E.-H.); ramadi.b@uaeu.ac.ae (B.a.-R.); 4 Departments of Pharmacology, College of Medicine & Health Sciences, United Arab Emirates University, Al Ain P.O. Box 17666, United Arab Emirates; shreeshojha@uaeu.ac.ae; 5 Zayed Centre for Health Sciences, United Arab Emirates University, Al Ain P.O. Box 17666, United Arab Emirates

**Keywords:** diabetes, pancreas, neural nitric oxide synthase, beta cell, oxidative stress, TBARS, ROS, glutathione reductase

## Abstract

Nitric oxide is generated from nitric oxide synthase following hyperglycemia-induced oxidative stress during the course of diabetes mellitus (DM). We examined the temporal immuno-expression of neuronal nitric oxide synthase (nNOS) in the pancreas of diabetic and non-diabetic rats using immunohistochemical, immunofluorescence and western blot techniques 12 h, 24 h, 1 week, 2 weeks, 1, 8 and 15 months after induction of DM. nNOS co-localized with pancreatic beta cells but disappears 12 h after the onset of DM. In contrast, the nNOS content of pancreatic nerves increased significantly (*p* < 0.001) 24 h after the induction of DM, and decreased sharply thereafter. However, nNOS-positive ganglion cells were observed even 15 months post-diabetes. ROS increased by more than 100% two months after the onset of DM compared to non-diabetic control but was significantly (*p* < 0.000001) reduced at 9 months after the induction of DM. The pancreatic content of GSH increased significantly (*p* < 0.02) after 9 months of DM. Although, TBARS content was significantly (*p* < 0.009; *p* < 0.002) lower in aged (9 months) non-diabetic and DM rats, TBARS rate was markedly (*p* < 0.02) higher 9 months after the induction of DM when compared to younger age group. In conclusion, nNOS is present in pancreatic beta cell, but disappears 12 h after the onset of diabetes. In contrast, the tissue level of nNOS of pancreatic nerves increased in the first week of diabetes, followed by a sharp reduction. nNOS may play important roles in the metabolism of pancreatic beta cell.

## 1. Introduction

Diabetes mellitus (DM) is a common, chronic endocrine disorder affecting millions of people worldwide. It is now estimated that currently, more than 537 million people between the age of 20 and 79 years have DM. This prevalence is projected to reach an alarming number of 783 million in the year 2045 [1]. DM is caused by inadequate secretion or effect of insulin. It can be inherited or caused by environmental factors, such as destruction of the pancreatic beta cell, viral infection, antibodies, inflammatory cytokines or any toxic agent that may destroy the insulin-producing pancreatic beta cells [2]. DM is associated with abnormal lipid, carbohydrate and protein metabolism [2]. One of the key cardinal points of DM is hyperglycemia, which occurs because there is not enough insulin to assist glucose transport into cells such as skeletal muscle cells, adipocytes and hepatocytes [3]. This diabetes-induced hyperglycemia is known to be associated with increased reactive oxygen species (ROS) production [4], which may overwhelm the capability of the endogenous antioxidant system, leading to oxidative stress. Increased oxidative stress level leads to the development and progression of micro- and macrovascular diseases affecting the retina, heart, kidney, nerves and many other organs [2,5]. One of the main molecules involved in the generation of cell and tissue oxidants is nitric oxide (NO).

NO, a short-lived radical gas, is involved in the regulation of several vital functions including vascular tone, hemodynamic states, coagulation cascade and immune functions [6,7,8,9]. This colorless, gaseous signaling molecule has also been implicated in stem cell biology where they stimulate stem cell lineages towards a specific cell type, thus facilitating the development of the embryo [10]. It is also a cellular bioproduct in a large variety of both animal and plant cells [11]. The plethora of important physiological and pathological processes involving NO, earned it the award of the “Molecule of the Year” in 1992 [12]. NO is produced from L-arginine, O_2_ and NADPH by nitric oxide synthase (NOS) [13]. There are three isoenzymes of NOS encoded by three separate genes, neuronal NOS (nNOS), endothelial NOS (eNOS) and cytokine-inducible NOS (iNOS). nNOS, the most notable isoform of NOS, is found first and foremost in the nervous system, where it is responsible for the production of NO 13]. In addition to its expression in neuronal elements, nNOS has also been observed in non-neuronal cells such as spermatocytes and Sertoli cells of the adult testis [14] and eosinophils [15] of connective tissues. nNOS has been demonstrated in the pancreas of rats [16] and other several mammalian species including the pig [17] and sheep [18]. nNOS has also been observed in human pancreatic beta cell line CM (malignant insulinoma) and rat insulinoma (INS-1) cell lines [19]. Recent studies, which show that nNOS co-localizes with pancreatic beta cells suggests that it may play a role in the control insulin secretion [16,20]. How does the pancreatic level of nNOS change after the onset of diabetes mellitus (DM)? The answer to this question has not yet been deciphered. Since diabetes has been shown to induce oxidative stress, the aim of this study was to determine whether diabetes induced by streptozotocin would cause any changes in the tissue content of nNOS in the rat pancreas.

Markers [reactive oxygen species (ROS), glutathione (GSH) and thiobarbituric acid reactive substances (TBARS)] of oxidative stress were also examined in the pancreas of diabetic rats. Oxidative stress has long been considered to play a role in the development of and severity of diabetes mellitus [21,22,23]. DM causes hyperglycemia, which has been shown to be a major inducer of oxidative stress [24,25]. This process puts a lot of pressure on the function of the endocrine pancreas. Reactive oxygen species cause glycation of proteins, depletion of NADPH through the polyol pathway and glucose autoxidation [23,26]. In normal tissues, endogenous antioxidants molecules destroy reactive molecular species to reduce oxidative stress [27,28,29], though in DM, hyperglycemia, a hallmark of diabetes, stimulates the production of reactive molecular species and oxidative stress, which destroy pancreatic beta cells [29].

How is nNOS related to ROS and other biomarkers of oxidative stress? The NO released by nNOS combines with superoxide to form peroxynitrite (O_2_•− + NO•→ONO_2_^−^). The superoxide substrate that combines with NO is generated in part by xanthine oxidase, reduced nicotinamide adenine dinucleotide phosphate (NADPH), cyclooxygenase and mitochondrial electron transport pathway. The superoxide ion can also be converted to hydrogen peroxide and oxygen by superoxide dismutase. However, hydrogen peroxide can be neutralized by both catalase and glutathione reductase, using glutathione as a substrate [30]. Both peroxynitrite and hydrogen peroxide can destroy any cell including pancreatic beta cell by inducing apoptosis [30]. In addition, the peroxynitrite formed from the combination of superoxide and nitric oxide may induce oxidation and nitration of proteins and lipid peroxidation, leading to inactivation of several enzymes [31] and other macromolecules. Malondialdehyde, an end product of lipid peroxidation, is measured using TBARS. The level of TBARS corresponds directly with the level of malondialdehyde and therefore reflect the degree of oxidative stress in cells and tissues [32].

In addition to the potential changes in the pancreatic level of nNOS, how does other biomarkers (ROS, GSH and TBARS) of oxidative stress change with time after the onset of diabetes? We intend to examine the changes in the tissue content of nNOS, ROS, TBARS and GSH in the rat pancreas after the onset of streptozotocin-induced DM in rats. If these changes were to occur, in what manner and duration do they appear?

## 2. Results

### 2.1. Weight and Blood Glucose Levels of Normal and Diabetic Rats

The weight and blood glucose of age-matched control and diabetic rats taken at the end of the experimental period are shown in Table 1. The results showed that both diabetic and control rats put on weight during the experimental period, but the age-matched non-diabetic control rats gained significantly (*p* < 0.004) more weight than diabetic rats. Moreover, diabetic rats had significantly (*p* < 0.0005) elevated blood glucose compared to controls.

### 2.2. Immunohistochemistry

**Figure 1:**
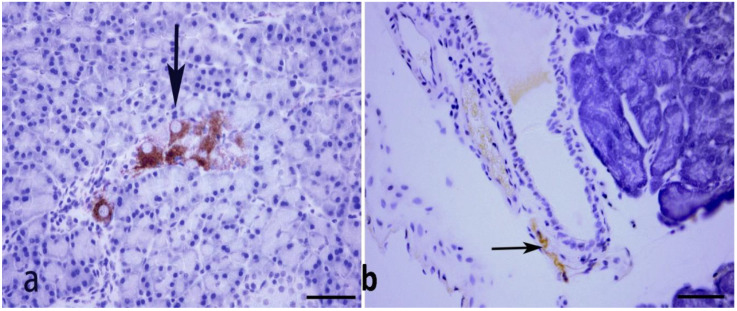
Shows nNOS-positive ganglion cells (thick arrow) and nerve fibres (thin arrow) in the pancreas of age-matched non-diabetic normal rat (**a**) and in pancreatic tissue fragments obtained 9 months after the onset of diabetes (**b**). nNOS-immunoreactive nerves are still present in the pancreas of diabetic rats (n = 6). Scale bar = 50 μm.

We performed immunohistochemistry and Western blot to determine whether nNOS is present in the animal model of our study. Immunohistochemical study showed that nerve fibers and ganglion cells of the pancreas contain nNOS, the isoform of nitric oxide synthase found mainly in nervous and skeletal tissues. nNOS was not detected immunohistochemically in pancreatic acinar cells (Figure 1). nNOS-immunoreactive nerves were also seen in the wall of pancreatic ducts (Figure 1).

### 2.3. Western Blot

**Figure 2:**
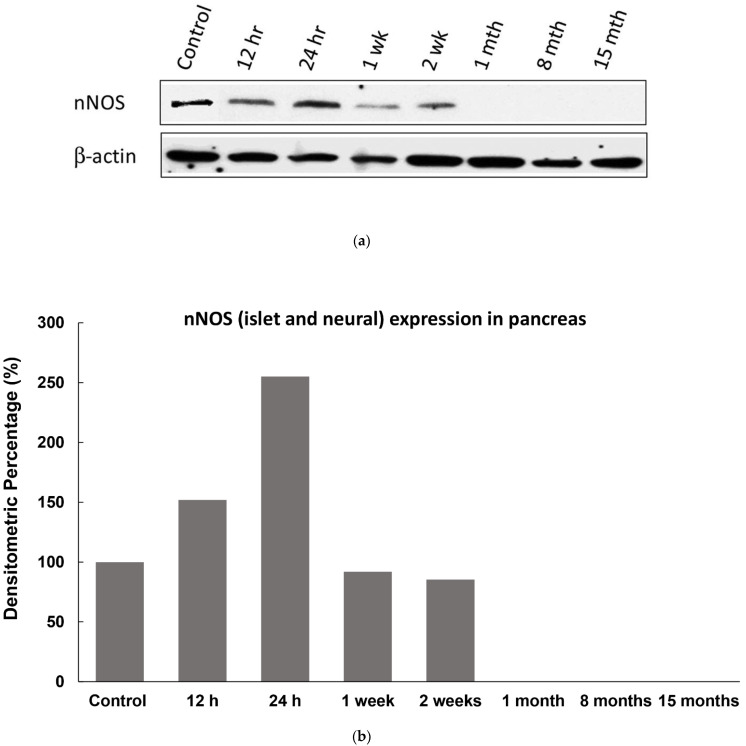

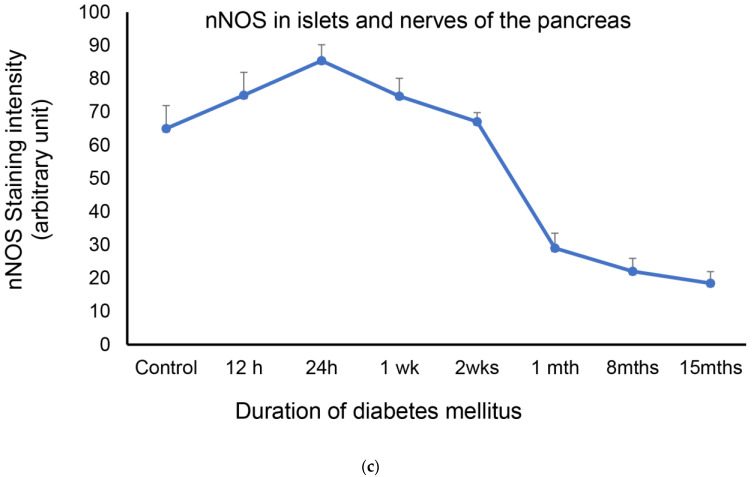
(**a**) Western blot image showing pancreatic (islets and pancreatic nerves) contents of nNOS at different stages of diabetes mellitus. Note the increase in nNOS content reached its peak 24 h after the induction of diabetes. The pancreatic tissue level decreased gradually after reaching its peak. (**b**) Densitometric analysis of the Western blot is shown in (**a**). (**c**) Shows the immunofluorescence staining intensity of nNOS in the pancreas (islets and pancreatic nerves) over the experimental period. Note the peak of gradual increase in nNOS, 24 h after the onset of diabetes. (n = 6).

We hypothesized that temporal changes occur in the tissue level of nNOS after the onset of diabetes mellitus. In order to examine these changes, we assessed the immune-expression of nNOS by Western blot in 60 µg of total protein from pancreatic tissue fragments of normal and STZ-diabetic rats using anti-nNOS antibody at 12 h, 24 h, 1 week, 2 weeks, 1, 8 and 15 months after the onset of diabetes when compared to non-diabetic control. The pancreatic tissue level of nNOS increased significantly 12 h and 24 h after the onset of diabetes. This increase was followed by a significant reduction that continued in weeks 1 and 2, followed by a larger decrease that was not detected at subsequent time (1, 8, 15 months) points (Figure 2a). The pancreatic tissue level of nNOS shows an acute increase after the onset of diabetes. The pancreatic level of nNOS went from the control timepoint to its highest point in 24 h, and declined gradually thereafter (Figure 2a–c).

### 2.4. Immunofluorescence Study of Insulin and nNOS

#### 2.4.1. Hours after the Onset of Diabetes Mellitus

**Figure 3:**
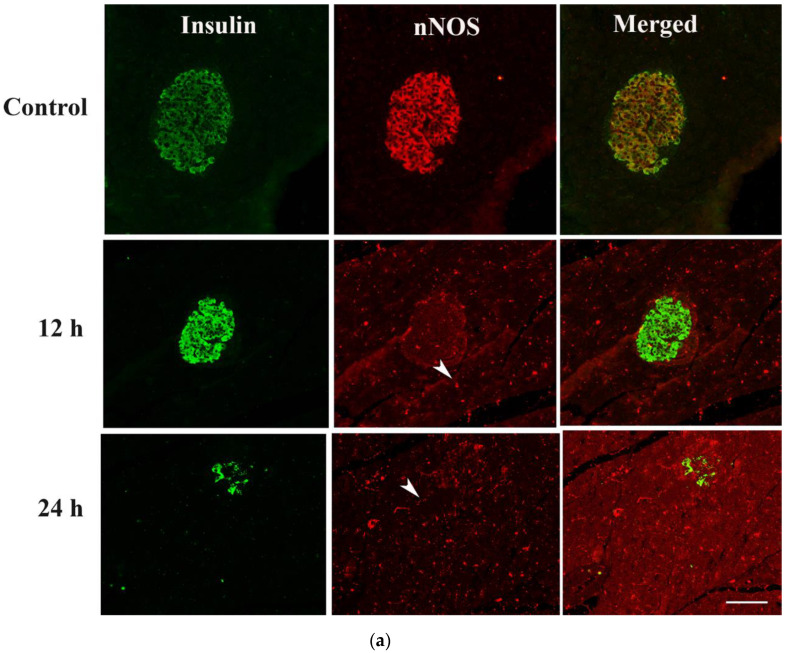

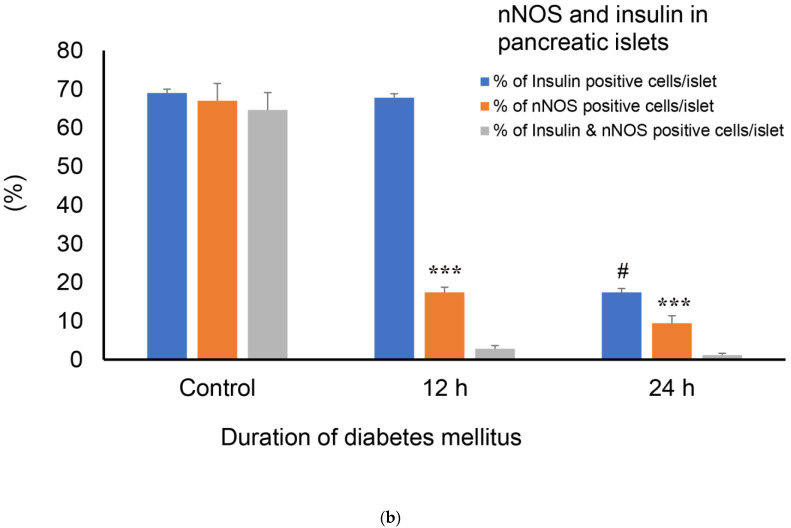
(**a**) shows insulin- (green) and nNOS-(red)-immuno-positive cells in rat islet of Langerhans of control and 12 h and 24 h after induction of diabetes. Note that nNOS co-localized (yellow color of merged image) with insulin in the islet of Langerhans in control, non-diabetic rats. No co-localization was observed after the induction of diabetes. nNOS-immuno-reactive nerve fibres (arrow head) were observed in both the endocrine and exocrine pancreas. Scale bar = 10 μm. (**b**) shows morphometric analysis of insulin- and nNOS-positive cells in the islet cells of the endocrine pancreas. Note that most of the insulin-containing cells in the control rat pancreas also contain nNOS. However, the induction of diabetes was associated with a significant (*** *p* < 0.001) decrease in the number of nNOS-positive cells in the islet cells of the endocrine pancreas. The number of insulin-positive cells in pancreatic islets also decreased markedly (# *p*< 0.001 when compared to those of control rats (n = 6)).

Double-labelled immunofluorescence study was performed to determine whether nNOS co-localizes with either insulin, and if yes, to what extent. nNOS co-localizes with insulin in pancreatic islet cells in control, non-diabetic rats, where most of the insulin-producing beta cells contain nNOS (Figure 3a). However, 12 h after the induction of diabetes, the number of nNOS-containing islet decreased significantly, albeit with more conspicuous nNOS-containing nerve profiles. Both insulin- as well as nNOS-containing endocrine cells were almost absent 24 h after the onset of diabetes. nNOS-immunoreactive nerves were now more prominent, especially 12 h and 24 h post-induction of diabetes (Figure 3a,b).

#### 2.4.2. Weeks after the Induction of Diabetes

**Figure 4:**
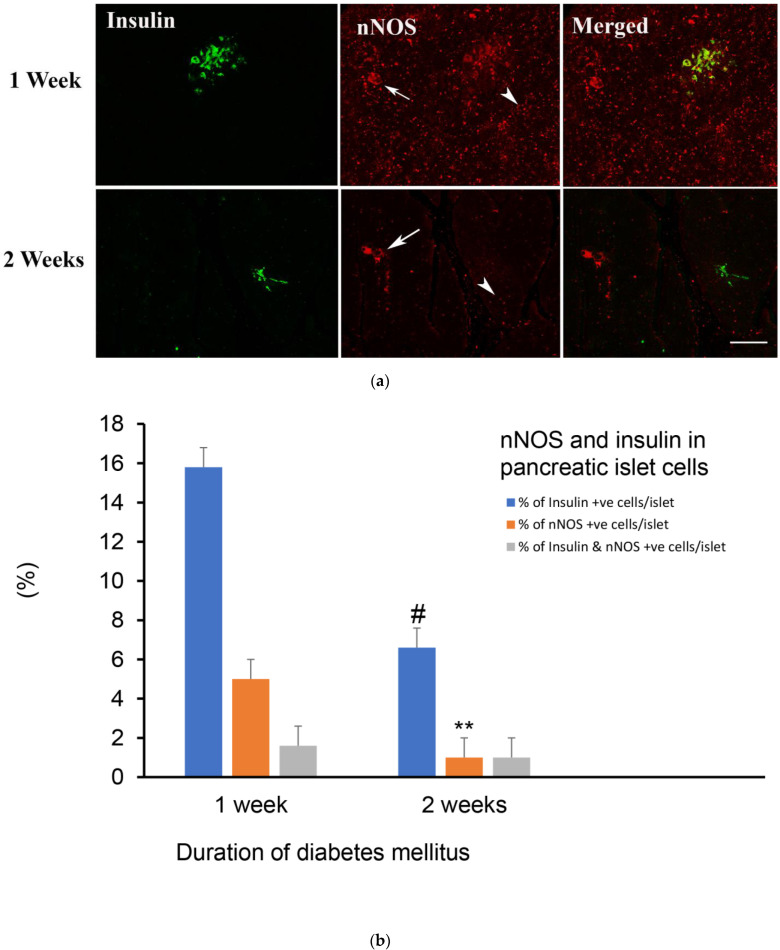
(**a**) shows insulin-(green) and nNOS-(red)-immuno-positive cells in the islet of Langerhans of rats 1 and 2 weeks after induction of diabetes. No significant co-localization between nNOS and insulin was observed after the induction of diabetes in week 2. nNOS-immunoreactive ganglion cell (arrow) and nerve fibres (arrow head) were, however, observed in the exocrine pancreas. Scale bar = 10 μm. (**b**) shows morphometric analysis of insulin- and nNOS positive cells in the islet cells of the endocrine pancreas. Note the significant reduction in the number of nNOS-positive cells (** *p* < 0.01) and insulin-immunopositive cells (# *p* < 0.01) in pancreatic islets in week 2 compared to week 1. (n = 6).

One and two weeks after the induction of diabetes with streptozotocin, the number and the size of pancreatic islets have significantly diminished. The islets contain few insulin- and nNOS-positive cells. The few insulin-positive cells also contain nNOS. At the end of the second week, the number of insulin-immunoreactive cells has experienced further decline. In contrast, nNOS-immunopositive varicose nerve fibres and ganglion cells were discernible in the pancreas (Figure 4a,b).

#### 2.4.3. Months after the Onset of Diabetes

**Figure 5:**
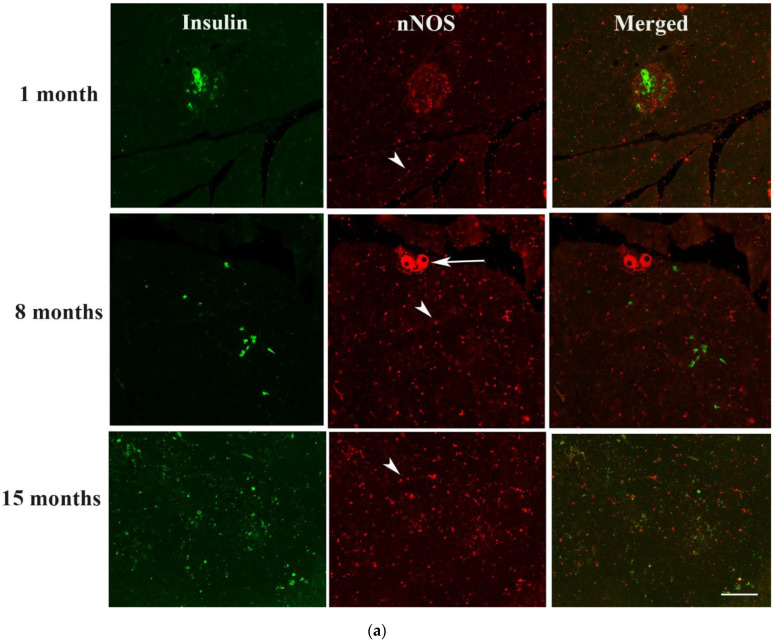

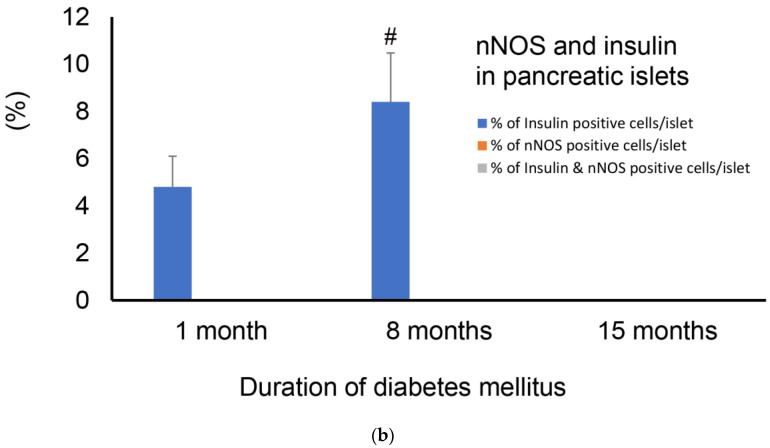
(**a**) shows insulin-(green) and nNOS-(red)-immuno-positive cells in the islet of Langerhans of rats 1, 8 and 15 months after induction of diabetes. No co-localization between nNOS and insulin was observed after the induction of diabetes. nNOS-immunoreactive ganglion cell (arrow) and nerve fibres (arrow head) were observed in the exocrine pancreas. Scale bar = 10 μm. (**b**) shows morphometric analysis of the insulin-positive cells in the islet cells of the endocrine pancreas. Note the slight but significant (# *p* < 0.01) increase in the number of insulin-containing cells after 8 months of diabetes compared to 1 month. (n = 6).

Only a few pancreatic beta cells could be observed 1 and 8 months after the induction of diabetes. Few nNOS-positive cell fragments were observed 1 month after the onset of diabetes, however, most of the islets have completely degenerated after 8 and 15 months of diabetes. Insulin- and nNOS-positive cells were not observed in islet cells after 15 months of diabetes. In contrast, nNOS-immunoreactive ganglion cells and nerves were discernible even after 15 months of DM (Figure 5).

### 2.5. Immunofluorescence Study of Glucagon and nNOS

A double-labelled immunofluorescence study was conducted to determine whether glucagon co-localizes with nNOS in the pancreatic alpha cell, the second most common cell in the islet of Langerhans. We conducted immunostaining similar to that of insulin versus nNOS.

#### 2.5.1. Hours after the Onset of Diabetes Mellitus

**Figure 6:**
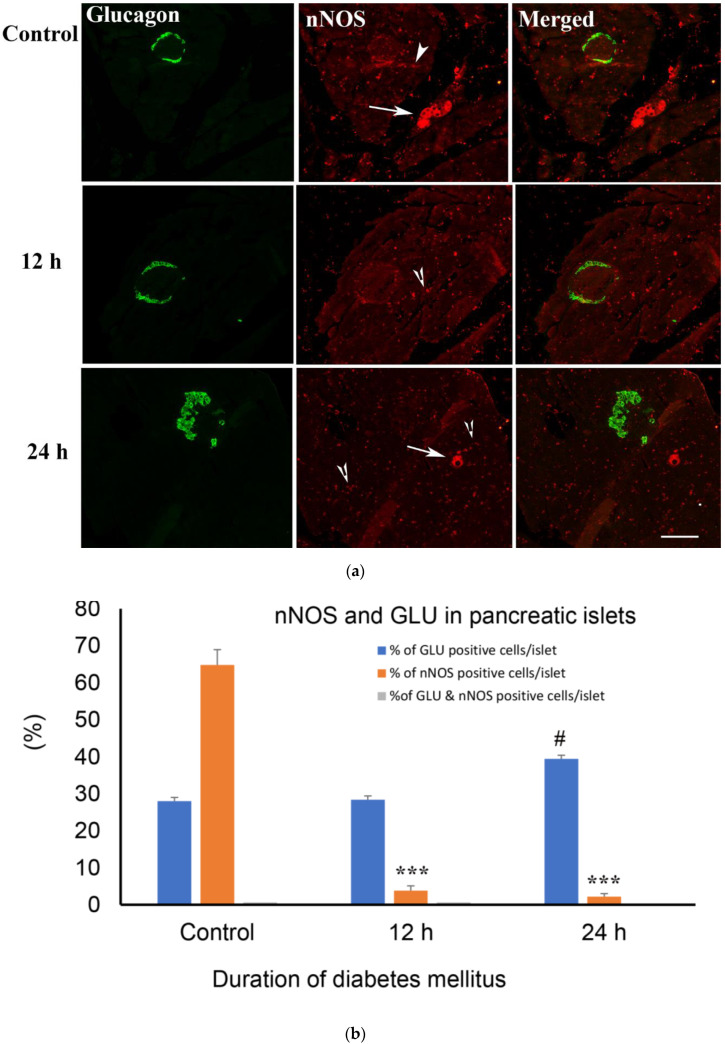
(**a**) shows glucagon (GLU)-(green) and nNOS-(red)-immuno-positive cells in the islet of Langerhans of rats of control and those with 12 h and 24 h of diabetes. There was no co-localization between glucagon and nNOS. nNOS-immunoreactive ganglion cell (arrow) and varicose nerve fibres (arrow head) were observed in the exocrine pancreas. Note the significant increase in the number of nNOS-containing nerve profiles after 12 and 24 h of diabetes. Scale bar = 10 μm. (**b**) shows morphometric analysis of glucagon-positive cells in the islet cells of the endocrine pancreas. There was a significant (*** *p*< 0.001) reduction in the number of nNOS-immunoreative cells in the islet of Langerhans compared to control. The number of glucagon-positive cells in the islets increased markedly (# *p* < 0.01) 24 h after the induction of diabetes, compared to the control (n = 6).

Diabetes, even for an acute, 24 h period of time, is associated with almost a complete loss of nNOS in pancreatic islet cells, but with a concomitant increase in the intensity of nNOS-positive nerve profiles in both the endocrine and exocrine pancreas. At 24 h after the induction of diabetes, the number of glucagon-positive cells has significantly increased. There was no significant sign of co-localization between glucagon and nNOS (Figure 6a,b).

#### 2.5.2. Weeks after the Induction of Diabetes Mellitus

**Figure 7:**
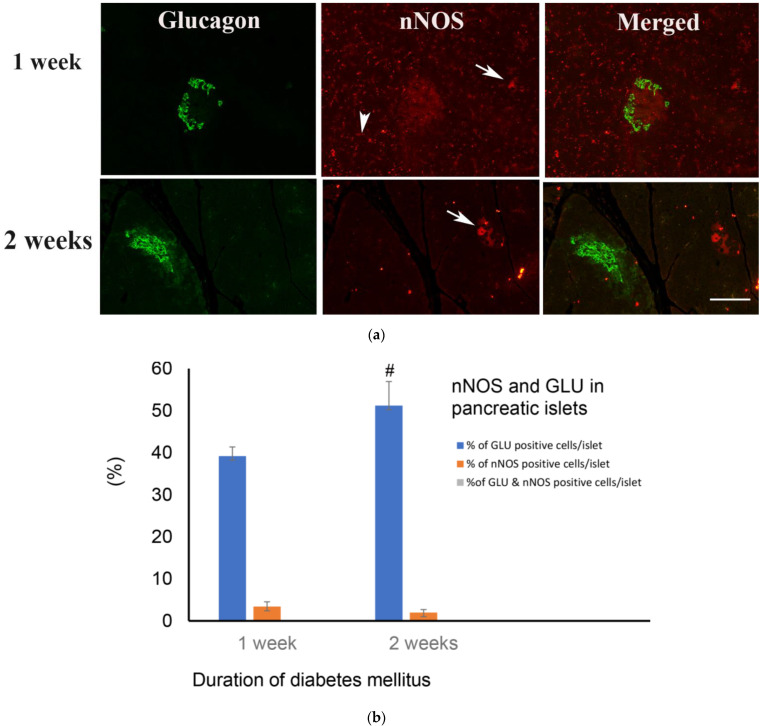
(**a**) shows glucagon (GLU)-(green) and nNOS-(red)-immuno-positive cells in pancreatic islets of rats 1 and 2 weeks after the onset of diabetes. There was no co-localization between glucagon and nNOS. nNOS-immunoreactive ganglion cell (arrow) and nerve fibres (arrow head) were observed in the exocrine pancreas. Scale bar = 10 μm. (**b**) shows morphometric analysis of the glucagon-positive cells in the islet cells of the endocrine pancreas. Note the slight but significant (# *p* < 0.01) increase in the number of glucagon-positive cells 2 weeks after induction of diabetes, when compared to week 1 (n = 6).

The number of glucagon-positive cells continue to increase after 1 and 2 weeks of diabetes mellitus. Only fragments of nNOS-containing cells were observed in this stage of diabetes. However, nNOS-immunoreactive ganglion cells and varicose nerve fibres can be discerned 1 and 2 weeks into diabetes (Figure 7a,b).

#### 2.5.3. Months after the Onset of Diabetes Mellitus

**Figure 8:**
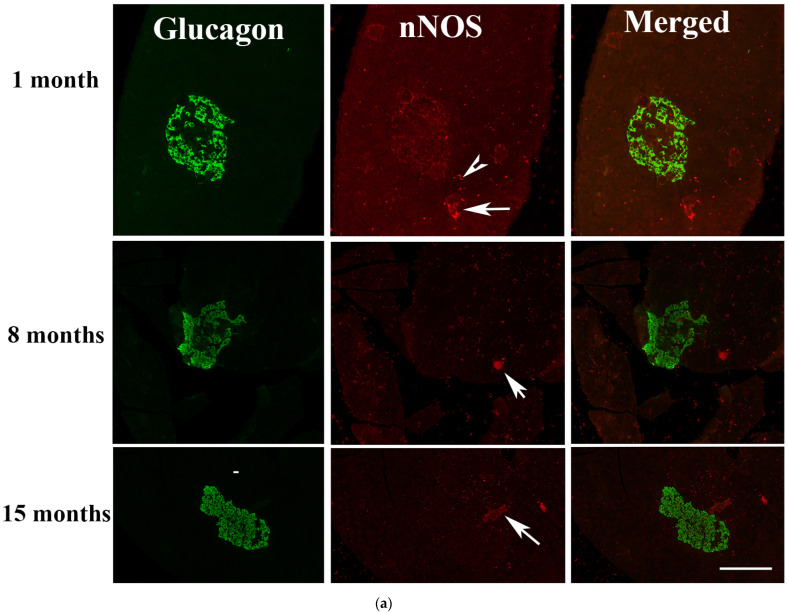

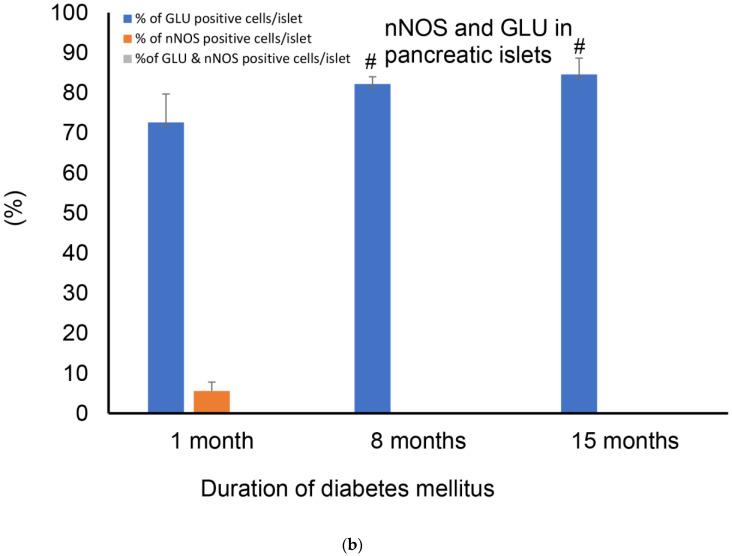
(**a**) shows glucagon (GLU)-(green) and nNOS-(red)-immuno-positive cells in pancreatic islets of rats 1, 8 and 15 months after the induction of diabetes. There was no co-localization between glucagon and nNOS. nNOS-immunoreactive ganglion cell (arrow) and varicose nerve fibres (arrow head) were observed in the exocrine pancreas. Fragments of nNOS-positive cells were observed in the islet 1 months after the onset of diabetes. Scale bar = 10 μm. (**b**) shows morphometric analysis of the glucagon-positive cells in the islet cells of the endocrine pancreas. Note the significant (# *p* < 0.01) increase in the number of glucagon-positive cells 8 and 15 months after the induction of diabetes. (n = 6).

One, eight and 15 months into diabetes witness significant changes in the pattern of distribution of both glucagon and nNOS. While only a residue of nNOS-positive were observed 1 month after the onset of diabetes, the pancreatic islet cells of rats with 8 and 15 months of diabetes have no significant nNOS-positive cells. In contrast, the number of glucagon-positive cells rose significantly (*p* < 0.001), with alpha cells almost occupying the whole islet. nNOS-immunoreactive ganglion cells persisted, and could be observed in the pancreas even after 15 months of DM (Figure 8a,b).

### 2.6. Measurement of Thiobarbituric Acid Reactive Substances (TBARS), Rate of TBARS, Reactive Oxygen Species (ROS) and Glutathione (GSH)

**Figure 9:**
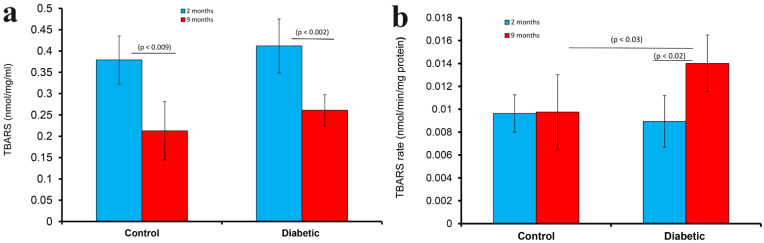
Histogram showing thiobarbituric acid reactive substances (TBARS) in the pancreas tissue fragments of diabetic rats 2 (blue) and 9 months (red) after the onset of diabetes. TBARS was also determined in age-matched controls. TBARS was significantly (*p* < 0.002) higher in the pancreas of rats with 2-month-old diabetes compared to that of 9-month-old rats. The level of TBARS was significantly (*p* < 0.009) higher in the pancreas of younger (2-month-old) rats compared to aged (9-month-old) in non-diabetic conditions (**a**). Histogram showing rate of TBARS in the pancreas of diabetic rats 2 (blue) and 9 months (red) after the onset of diabetes is shown in (**b**). Rate of TBARS was also determined in age-matched controls. The rate of TBARS was significantly (*p* < 0.03) higher after the onset of diabetes in the older age group. The rate of TBARS was significantly (*p* < 0.02) higher in the pancreas of rat with 9-month-old of diabetes, compared to those with a shorter period of diabetes (**b**).

TBARS was significantly (*p* < 0.002) higher in the pancreas of rats with 2-month-old diabetes compared to 9-month-old rats. In a similar trend, the tissue level of TBARS was significantly (*p* < 0.009) higher in the pancreas of younger (2-month-old) non-diabetic rats compared to that of 9-month-old non-diabetic rats (Figure 9a).

In normal, non-diabetic rats, the rate TBARS production in the pancreas was not significantly different when young (2-month-old) rats are compared to old (9-month) rats. However, the rate of TBARS production was significantly (*p* < 0.02) higher 9 months after the onset of diabetes when compared to non-diabetic rats, and to those rats with diabetes of shorter duration (Figure 9b). However, TBARS rate was significantly (*p* < 0.03) higher in diabetic (9-month) compared to corresponding age-matched controls.

### 2.7. Reactive Oxygen Species (ROS)

**Figure 10:**
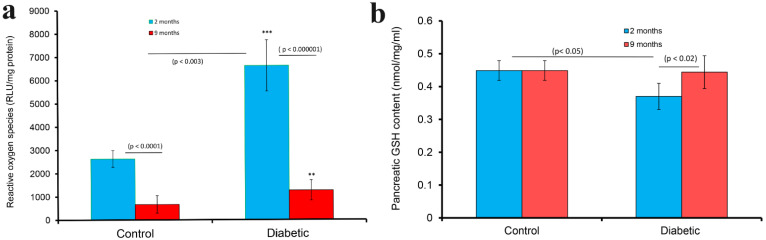
(**a**) shows histogram of reactive oxygen species (ROS) in the pancreas of diabetic rats 2 (blue) and 9 months (red) after the onset of diabetes. ROS was also determined in age-matched controls. ROS was significantly (*** *p* < 0.004) higher after the onset of diabetes lasting 2 months. ROS was significantly (** *p* < 0.03) higher after the induction of diabetes that lasted 9 months but less dramatic compared to those of diabetes of a shorter duration. ROS was significantly higher in the pancreas of 2-month-old rats in both normal (*p* < 0.0001) and diabetic (*p* < 0.000001) conditions compared to that of 9-month-old rats (**a**). (**b**) shows the histogram of glutathione (GSH) in the pancreas of diabetic rats 2 (blue) and 9 months (red) after the onset of diabetes. GSH was also determined in age-matched controls. While the GSH level of pancreatic tissues was significantly (*p* < 0.05) reduced 2 months after the induction of diabetes, that of 9-month-old rats approached control level. The pancreatic GSH content of rats with 9-month-old diabetes was significantly (*p* < 0.02) higher than those with 2-month-old diabetes (n = 4).

Reactive oxygen species (ROS) was significantly (*p* < 0.0001) lower in the pancreas of aged (9 month), non-diabetic rats when compared to that of younger (2 month), non-diabetic rats. In a similar trend, the pancreatic ROS level rose markedly (*p* < 0.003) after the induction of diabetes. However, the pancreatic ROS level was significantly (*p* < 0.000001) lower 9 months after the onset of DM compared to 2-month-old group (Figure 10a).

The pancreatic tissue level of glutathione (GSH) was similar in 2 and 9 months old non-diabetic rats. However, the GSH level of pancreatic tissues was significantly (*p* < 0.05) reduced 2 months after the induction of diabetes. Pancreatic GSH level rose to normal level after 9 months of DM, and was significantly (*p* < 0.02) higher than that of the 2-month-old group (Figure 10b).

## 3. Discussion

Neuronal nitric oxide synthase (nNOS), the enzyme that converts L-arginine to NO, has already been shown to be present in the pancreas of normal pigs [17] and sheep [18]. Indeed, nNOS has been localized to pancreatic beta cells and in insulin-producing cell lines [16,19,20]. It should not be surprising to find nNOS in pancreatic beta cells, as many neuropeptides and or neurotransmitters have been observed in pancreatic beta cells. These neuropeptides and neurotransmitters include ghrelin [33,34], gamma aminobutyric acid [35], resistin [36], orexins [37], galanin [38], nociceptin [39,40], calcitonin gene-related peptide [41] and many others. The possible reason for the detection of neuropeptides and neurotransmitters in pancreatic islet cells include the fact that pancreatic beta cells are phylogenetically similar to that of neurons, which developed from the neural crest [42,43]. We also showed that nNOS is present in pancreatic ganglion cells of normal rats. This observation corroborates other findings that nNOS is indeed present in ganglion cells of the pancreas [18,44], as well as those in the enteric nervous system of non-diabetic [45] and diabetic [45] rodents. nNOS generates most of the NO needed for the normal function of the gastrointestinal system. NO adds inhibitory signals to gastric movement by initiating smooth muscle relaxation [46]. NO synthesized by nNOS is also involved in the protection of the gastric mucosa against NSAID-induced damage [47].

### 3.1. nNOS in Pancreatic Beta Cell

In addition to its presence in pancreatic nerve profiles, nNOS has also been previously reported in pancreatic islet cells of normal rats [16]. Our results showing the presence of nNOS in the pancreas of normal rat islets corroborates previously reported data [16]. However, most of the studies performed on the presence of nNOS in the endocrine pancreas were undertaken in normal rodent models. Here, we showed that nNOS disappears almost completely from pancreatic beta cells 12 h after the induction of diabetes mellitus. The reason for this phenomenon is not clear. The reason why nNOS disappears from the pancreatic beta cells after the onset of diabetes may be because nNOS is highly sensitive to streptozotocin, the chemical agent used in the induction of diabetes. The disappearance may also be due to changes in the chemical constituents of the beta cell after the induction of diabetes. It has been shown that streptozotocin causes several changes to the beta cell, including viability, ability to secrete insulin and many intracellular components of the cell [48]. 

What function does nNOS have in the pancreas? Previous reports have indicated a dual role for nNOS in pancreatic beta cells [49]. One of the functions of the NO released by nNOS during exposure to cytokines is to induce the destruction of pancreatic beta cell. In contrast to this function, L-arginine, a substrate for NO production, plays a role in the process leading to insulin release from pancreatic beta cell [49,50,51,52]. All of these data suggest that nNOS has indeed an important role to play in the function of the endocrine pancreas.

### 3.2. nNOS in Pancreatic Nerve Profiles

In contrast to the loss of nNOS in the pancreatic beta cells of the endocrine pancreas, the pancreatic tissue level of nNOS increased significantly 12 h and 24 h after the induction of diabetes and remained so for 2 weeks. Thereafter, the pancreatic tissue content of nNOS decreased sharply after these time points. This notable increase in nNOS in the pancreas was mostly from the contribution of nNOS localized to varicose nerve terminals and ganglion cells of the pancreas. This observation supports previous findings from our laboratory that the presence of nNOS-positive neurons innervating the gastroduodenal tract still persist after the onset of both short- and long-term diabetes [45]. To the best of our knowledge, this is the first study to examine the tissue level of nNOS in the pancreas at several time points after the onset of diabetes, and it is therefore difficult to compare this result with those of the literature. However, the short-term increase in the pancreatic neural tissue level of nNOS may be due to the generation of oxidative stress by diabetes-induced hyperglycemia. It is also well known that diabetes, via hyperglycemia, induces reactive oxygen species [21,22,23]. Moreover, the increase in the level of nNOS in the nerve profiles of the diabetic rat pancreas may also be attributed to the toxic effect of STZ, since NO production has been reported to increase in STZ-induced oxidative stress [52,53,54]. One month after the induction of diabetes, the pancreatic level of nNOS decreased significantly. This observation may be attributed to the continued oxidative stress that may have destroyed the capability of pancreatic nerves to produce nNOS, irrespective of the fact that an increase in tissue GSH was observed. The increase in GSH was probably not enough to offset the overall harmful effect of oxidative stress. Although a progressive increase in the pancreatic content of nNOS in diabetic rats has not been described, other investigators have demonstrated a decrease in the number of nNOS-positive nerves in the gastrointestinal tract (antrum) of diabetic rats [53,55] and impaired NOS synthesis in diabetes [56]. In contrast to these literature reports on the content of nNOS in the enteric nervous system, results from our laboratory showed a decrease in the size of nNOS-immunoreative myenteric ganglion cells and increased nNOS content four weeks after the induction of diabetes [45]. The observation on the initial increase, followed by a decrease in the pancreatic tissue level of nNOS after the onset of diabetes, is supported by an increase in the rate of TBARS and tissue levels of ROS, markers of oxidative stress that were observed in this study.

### 3.3. Markers of Oxidative Stress

The pancreatic tissue level of ROS was markedly increased after the onset of diabetes, indicating the role of oxidative stress in the changes observed in nNOS immune-expression in the pancreas. Moreover, the pancreatic content of these markers of oxidative stress was age-dependent, as the tissue levels of these markers were more pronounced in pancreatic tissue after the onset of short term diabetes. This observation corroborates those reported in the literature [57,58,59]. It was of interest to note that ROS production was still significantly high 2 months after the onset of DM. However, the ROS level in the pancreas of rats 9 months after the induction of DM was significantly lower compared to that of rats with DM of a 2-month duration. This may indicate that the time to intervene in the suppression of ROS would be within 2 months of DM. The increase in ROS in the pancreas of rats with 2 months’ duration is associated with increased TBARS (increased lipid peroxidation). The marked reduction in the level of GSH may point to the fact that this endogenous antioxidant has almost been used up to reduce (fight) the oxidative stress observed at this stage of DM

## 4. Materials and Methods

### 4.1. Experimental Animals and Induction of Diabetes Mellitus

Male Wistar rats, aged 12 weeks and weighing approximately 250 gm were used in this study. Wistar rats were obtained from the United Arab Emirates University breeding colony. All experimental protocols and study procedures were performed in accordance with the guidelines set by the National Institute of Health (NIH) for the care and use of laboratory animals and approved by the Animal Research Ethics Committee of the College of Medicine and Health Sciences, UAE University (A5–14).

The rats were divided into two groups; STZ-induced diabetics and age-matched controls. The experimental animals were sacrificed at 12 h, 24 h, 1 week, 2 weeks, 1, 2, 8, 9 and 15 months after induction of diabetes. Rat were made diabetic by a single intraperitoneal injection of STZ (Sigma, Poole, UK) at 60 mg kg^−1^ prepared in 5 mM citrate buffer, pH 4.50 [60,61]. The animals were placed in plastic cages and maintained on standard laboratory animal diet and water ad libitum. Blood glucose was measured for each individual animal with One-Touch II^®^ Glucometer (LifeScan, Johnson and Johnson, Milpitas, CA, USA). Rats were considered diabetic if the random blood glucose levels were equal to or more than 300 mg dl^−1^. Rats were weighed at the end of the experimental period. There were 6 rats for each time point (n = 6).

### 4.2. Immunohistochemistry

Pancreatic tissue fragments of normal and diabetic rat pancreas were fixed quickly in Zamboni’s solution, embedded in paraffin, sectioned and processed for immunohistochemistry according to that reported by Adeghate and Donáth and Adeghate [62,63]. Briefly, 6 µm-thick tissue sections were made on a Shandon microtome (Shandon AS325, Pittsburgh, PA, USA), placed in a water bath at 48 °C, transferred onto prewashed microscopic slides and later de-paraffinized in xylene. The sections were later incubated in anti-nNOS serum (1:1000 Transduction Laboratories, Lexington, KY, USA) and a negative control reagent for 24 h at 4 °C. The slides were later rinsed and incubated for 30 min with prediluted biotinylated anti-mouse IgG, rinsed in Tris buffered saline (TBS) before incubation with streptavidin peroxidase conjugate for 45 min. Sites of peroxidase activity was detected by 3,3-diaminobenzidine tetrahydrochloride and 0.03% H_2_O_2_. The sections were counter-stained with hematoxylin, dehydrated and mounted in Cytoseal 60^®^ (Stephens Scientific, Riverdale, NJ, USA). The specificity of the antibody was confirmed by processing tissue samples in the absence of anti-nNOS serum. No specific immunostaining was observed when anti-nNOS antibody was omitted.

### 4.3. Immunofluorescence

Pancreatic tissue fragments of normal and diabetic rat pancreas were processed for immunofluorescence according to previously reported method [64]. Briefly, 6 µm-thick tissue sections, cut on a Shandon microtome, were de-paraffinized in xylene before overnight incubation in anti-nNOS serum (1:500, Sigma-Aldrich, St. Louis, MO, USA) and negative control reagent for 24 h at 4 °C. The sections were later treated with immune conjugated TRITC, before incubation with either insulin or glucagon. Insulin and glucagon immunoreactive tissue sites were conjugated with FITC, washed in PBS and mounted with CITI-Fluore (Science Services GmbH, München, Germany). Fluorescence images were taken with AxioCam HRc digital camera using AxioVision 3.0 Software (Carl Zeiss, Oberkochen, Germany). Images were later analyzed with Image J version 1.8. The specificity of the antibody was further confirmed by processing tissue samples in the absence of anti-nNOS serum. No specific immunostaining was observed when either anti-nNOS, insulin and glucagon antibodies were omitted.

### 4.4. Measurement of Thiobarbituric Acid Reactive Substances (TBARS), Rate of TBARS, Reactive Oxygen Species (ROS), Glutathione (GSH)

The tissue levels of these markers of oxidative stress were measured to determine how different stages of STZ-induced diabetes and ageing affect the ability of the pancreas to defend itself from free radicals. Pancreatic tissue samples were taken from young (2 months) and (9 months) old rats to examine the impact of aging on selected markers of oxidative stress.

### 4.5. Lipid Peroxidation

The degree of lipid peroxidation was quantified by estimating TBARS level using malondialdehyde as standard [65]. The rate of TBARS estimated the formation of lipid peroxidation per second. Malondialdehyde is a well-characterized end-product of the oxidative degradation of lipid (lipid peroxidation). The more severe the oxidative stress, the more the lipid peroxidation and its products, such as malondialdehyde, indicating that many cell components, including cell membranes, will be damaged. In our study, we measured the reaction product of thiobarbituric acid with malondialdehyde to estimate the quantity of malondialdehyde formed in pancreatic tissue fragments [65].

### 4.6. Reactive Oxygen Species (ROS)

Reactive oxygen species was estimated as being equal to the direct formation of NAD(P)H oxidase-dependent lucigen-enhanced chemiluscence from freshly prepared mitochondria [22]. This is a measurement of intracellular superoxide ions released by cells using lucigen as a probe. The chemical reaction between superoxide and lucigen chemiluminescence, which is then quantified using chemiluminescence immunoassay [22]. The higher the level of ROS, the more severe the outcome of oxidative stress would be.

### 4.7. Glutathione (GSH)

The pancreatic tissue level of this endogenous antioxidant was determined as a protein-free sulfihydril content using the Ellman’s reagent [66]. GSH is an important endogenous antioxidant capable of protecting cells from the toxic effects of reactive oxygen species. It exerts its cytoprotective effect by neutralizing ROS to form glutathione disulfide [67].

### 4.8. Western Blot Analysis of Neural Nitric Oxide Synthase (nNOS)

Detailed procedures for the isolation of pancreatic tissue and immunoblotting have been published previously [68,69]. Pancreatic tissue fragments were briefly taken at 12 h, 24 h, 1 week, 2 week, 1, 8 and 15 months after the onset of diabetes. The tissue samples (n = 6/per group) were washed twice with cold phosphate buffered saline (PBS) and directly frozen in liquid nitrogen. Frozen pancreatic tissue fragments (1 to 2 g) were broken under liquid nitrogen with a ceramic mortar and pestle. Two volumes of extraction buffer [100 mM HEPES, pH 7.5, 10% sucrose, 10 mM DTT, 0.1% CHAPS, 150 mM NaCl and protease inhibitors (1 mM PMSF and 1 μg/mL of leupeptin, aprotonin and pepstatin A)] were added to the powder of broken pancreatic cells. The suspension was cleared by centrifugation at 14,000× *g* for 30 min. The supernatant was later retrieved and stored at −80 °C until analyzed. The protein concentration of cellular lysates was measured by the Bradford method using a colorimetric protein assay kit (BioRad, Irvine, CA, USA). Aliquots (60 μg) of total proteins were resolved on 8% SDS-PAGE, transferred to a PVDF membrane and blocked with 5% non-fat milk for 1 h. The blots were probed with 1:1000 dilution of mouse monoclonal anti-nNOS antibody (Transduction Laboratories, Lexington, KY, USA) in PBS containing 0.1% Tween-20 and 2% non-fat dry milk for 2 h at room temperature. Thereafter, the blots were treated with HRP-conjugated antibody against mouse IgG (1:2000 dilution; Sigma, Poole, UK) and the immunoreactive bands were visualized and analyzed using Super Signal West Pico chemiluminescent kit according to the supplier’s protocol (Pierce Chemical Co., Dallas, TX, USA). In order to confirm equal loading of proteins, the blots were also incubated with a polyclonal antibody against β-actin (Santa Cruz Biotechnology; 1:2500 dilution). Pancreatic tissue fragments obtained from age-matched controls were processed according to the above procedure. Densitometric analysis of the band intensity on the blots was done using ImageJ (National Institutes of Health, Bethesda, MD, USA), as described previously [70].

### 4.9. Densitometric Analysis of Intensity of nNOS Immunofluorescence

The density of nNOS immunofluorescence staining was analyzed with Image J software^®^ (NIH, Bethesda, MD, USA) according to a previously reported method [28]. Briefly, each nNOS stained image was copied to the computer clipboard and placed on 8-bit slot of Image J, after which the image was inverted. The total number of pixels were collected on the line tool. The peaks of the pixel were analyzed as percentages of the control image. The analysis was performed for 6 consecutive section and per group. Data were taken as mean ± SD.

## 5. Statistical Analysis

All data were taken as mean ± standard deviation. Differences among the study groups were analyzed with One-way ANOVA. *p* values were considered significant if less than 0.05.

## 6. Conclusions

In conclusion, our study showed that nNOS is present in both pancreatic islet cells and nerves of the pancreas. nNOS strongly co-localizes with pancreatic beta cells in the islet of Langerhans of normal, non-diabetic rats, but disappears after the onset of diabetes. However, the immuno-expression of nNOS is significantly increased in the nerves of diabetic rat pancreas, hours after the onset of diabetes, and reduces gradually with time. Markers of oxidative stress, including TBARS, rate of TBARS, ROS and GSH, are more pronounced in the pancreas of rats with a shorter duration of diabetes. Recognition of the temporal changes in the immuno-expression of nNOS and levels of biomarkers of oxidative stress may help in deciding the optimal time to intervene in hyperglycemia-induced oxidative stress.

## Figures and Tables

**Table 1:** Weight and blood glucose levels of normal and diabetic rats.

	Normal	Diabetic	Difference
Weight (g)	303.33 ± 14.2	244.67 ± 33.6	*p* < 0.004
Blood glucose (mg/dL)	55.9 ± 5.5	528.8 ± 55.14	*p* < 0.0005

Note that there is a significant decrease in weight and a marked elevation in blood glucose level of diabetic rats at the end of the experiment.

## Data Availability

The datasets obtained during and/or analyzed during the course of the current study are available in the manuscript.
